# Supplementary material for: Association between diet-related greenhouse gas emissions and nutrient intake adequacy among Japanese adults
Source: PLoS One. 2020 Oct 23;15(10):e0240803. doi: 10.1371/journal.pone.0240803 (PMC7584234; doi:10.1371/journal.pone.0240803)
Supplement: S1 File — (DOCX) [file pone.0240803.s001.docx]

# S1 Appendix: Questions for demographic variables

ID ( )

Age ( )

Sex ( men / women )

Occupation:

Please select one option that is most appropriate for your occupation and mark it.

If you are doing multiple jobs, choose the one that spends the most time.

□ Clerical (incl. counsellors and managers)

□ Nursing care (nursing staff, support staff, childcare staff, etc.)

□ Medical assistant (doctors, nurses, etc.)

□ Cooking assistant (incl. meal planning and preparation)

□ Others (Please tell me specifically)

Educational background:

Which of the following schools did you last graduate?

□ Junior high school

□ Senior high school

□ Vocational school or junior college

□ University or graduate school

□ Other

Smoking habit:

Do you smoke? If it has been less than a year since you quit smoking, check "Smoke" and answer the habit you had when you were smoking.

□ Never smoked

□ Quit smoking more than a year ago

□ Smoke (20 or less cigarettes per day)

□ Smoke (more than 20 cigarettes per day)

# S1 Table. Sex- and age-specific Diet reference intakes values for Japanese population^a^

|  | Male | | | | Female | | | |
| --- | --- | --- | --- | --- | --- | --- | --- | --- |
|  | 18-29 y | 30-49 y | 50-64 y | 65-74 y | 18-29 y | 30-49 y | 50-69 y | 65-74 y |
| Tentative Dietary Goal for Preventing Lifestyle-related Diseases | | |  |  |  |  |  |  |
| Protein (% energy) | 13-20 | 13-20 | 14-20 | 15-20 | 13-20 | 13-20 | 14-20 | 15-20 |
| Fat (% energy) | 20-30 | 20-30 | 20-30 | 20-30 | 20-30 | 20-30 | 20-30 | 20-30 |
| Saturated fat (% energy) | <7 | <7 | <7 | <7 | <7 | <7 | <7 | <7 |
| Carbohydrate (% energy) | 50-65 | 50-65 | 50-65 | 50-65 | 50-65 | 50-65 | 50-65 | 50-65 |
| Dietary fiber (g/d) | >21 | >21 | >21 | >20 | >18 | >18 | >18 | >17 |
| Sodium (g NaCl equivalent/d) | <7.5 | <7.5 | <7.5 | <7.5 | <6.5 | <6.5 | <6.5 | <6.5 |
| Potassium (mg/d) | 3000 | 3000 | 3000 | 3000 | 2600 | 2600 | 2600 | 2600 |
| Estimated Average Requirement |  |  |  |  |  |  |  |  |
| Protein (g/d) | 50 | 50 | 50 | 50 | 40 | 40 | 40 | 40 |
| Vitamin A (μg RAE/d) | 600 | 650 | 650 | 600 | 450 | 500 | 500 | 450 |
| Vitamin B-1 (mg/d) | 1.2 | 1.2 | 1.1 | 1.1 | 0.9 | 0.9 | 0.9 | 0.8 |
| Vitamin B-2 (mg/d) | 1.3 | 1.3 | 1.2 | 1.2 | 1.0 | 1.0 | 1.0 | 1.0 |
| Niacin (mg/d) | 13 | 13 | 12 | 12 | 9 | 10 | 9 | 9 |
| Vitamin B-6 (mg/d) | 1.1 | 1.1 | 1.1 | 1.1 | 1.0 | 1.0 | 1.0 | 1.0 |
| Vitamin B-12 (μg/d) | 2.0 | 2.0 | 2.0 | 2.0 | 2.0 | 2.0 | 2.0 | 2.0 |
| Folate (μg/d) | 200 | 200 | 200 | 200 | 200 | 200 | 200 | 200 |
| Vitamin C (mg/d) | 85 | 85 | 85 | 80 | 85 | 85 | 85 | 80 |
| Calcium (mg/d) | 650 | 550 | 600 | 600 | 550 | 550 | 550 | 550 |
| Magnesium (mg/d) | 280 | 310 | 310 | 290 | 230 | 240 | 240 | 230 |
| Iron (mg/d) | 6.5 | 6.5 | 6.5 | 6.0 | 8.5 | 9 | 5.5 | 5.0 |
| Zinc (mg/d) | 9 | 9 | 9 | 9 | 7 | 7 | 7 | 6 |
| Copper (mg/d) | 0.7 | 0.7 | 0.7 | 0.7 | 0.6 | 0.6 | 0.6 | 0.6 |

RAE, retinol activity equivalent.

^a^ Values are derived from Diet reference intakes for Japanese, 2020 [41].

# S2 Table. Basic characteristics of participants according to quartile (Q) of diet-related GHGE (g CO_2_-eq/day) among 196 Japanese men and 196 women (aged 20-69 y)^a^

|  | Men | | | | | | Women | | | | | |
| --- | --- | --- | --- | --- | --- | --- | --- | --- | --- | --- | --- | --- |
|  | All (n=196) | Q1 (n=49) | Q2 (n=49) | Q3 (n=49) | Q4 (n=49) |  | All (n=196) | Q1 (n=49) | Q2 (n=49) | Q3 (n=49) | Q4 (n=49) |  |
|  |  | 3550 (3399, 3673)^b^ | 4044 (3927, 4229)^b^ | 4511 (4397, 4617)^b^ | 5174 (4934, 5473)^b^ | P^c^ |  | 3031 (2840, 3204)^b^ | 3450 (3405, 3587)^b^ | 3844 (3781, 3955)^b^ | 4420 (4270, 4574)^b^ | P^c^ |
| Age (years) | 44.6 ± 13.3 | 39.4 ± 13.4* | 46.5 ± 13.3† | 44.1 ± 12.9*† | 48.7 ± 12.1† | 0.004 | 44.4 ± 13.5 | 39.9 ± 12.9* | 43.3 ± 13.9*† | 44.2 ± 13.4*† | 50.2 ± 11.8† | 0.002 |
| Body mass index (kg/m^2^) | 24.0 ± 3.5 | 23.5 ± 3.9 | 24.5 ± 4.0 | 23.9 ± 3.1 | 24.1 ± 2.9 | 0.60 | 22.6 ± 3.7 | 22.6 ± 3.8 | 22.7 ± 3.9 | 21.9 ± 2.8 | 23.3 ± 4.2 | 0.33 |
| Living area (%) |  |  |  |  |  | 0.07 |  |  |  |  |  | 0.02 |
| Hokkaido and Tohoku | 15.3 | 24.5 | 22.4 | 8.2 | 6.1 |  | 14.8 | 18.4 | 14.3 | 6.1 | 20.4 |  |
| Kanto | 20.4 | 12.2 | 26.5 | 24.5 | 18.4 |  | 19.9 | 28.6 | 22.4 | 20.4 | 8.2 |  |
| Hokuriku and Tokai | 9.2 | 12.2 | 10.2 | 8.2 | 6.1 |  | 9.7 | 8.2 | 10.2 | 16.3 | 4.1 |  |
| Kinki | 14.8 | 12.2 | 8.2 | 22.4 | 16.3 |  | 15.3 | 14.3 | 6.1 | 10.2 | 30.6 |  |
| Chugoku and Shikoku | 19.9 | 20.4 | 20.4 | 20.4 | 18.4 |  | 20.4 | 16.3 | 26.5 | 22.4 | 16.3 |  |
| Kyusyu and Okinawa | 20.4 | 18.4 | 12.2 | 16.3 | 34.7 |  | 19.9 | 14.3 | 20.4 | 24.5 | 20.4 |  |
| Occupation (%) |  |  |  |  |  | 0.177 |  |  |  |  |  | 0.002 |
| Clerical | 46.4 | 28.6 | 51.0 | 53.1 | 53.1 |  | 37.2 | 42.9 | 30.6 | 44.9 | 30.6 |  |
| Nursing care | 39.3 | 59.2 | 32.7 | 34.7 | 30.6 |  | 44.4 | 44.9 | 51.0 | 32.7 | 49.0 |  |
| Medical assistant | 2.0 | 2.0 | 0.0 | 4.1 | 2.0 |  | 4.1 | 2.0 | 0.0 | 14.3 | 0.0 |  |
| Cooking assistant | 3.1 | 2.0 | 6.1 | 2.0 | 2.0 |  | 9.2 | 6.1 | 8.2 | 4.1 | 18.4 |  |
| Others | 9.2 | 8.2 | 10.2 | 6.1 | 12.2 |  | 5.1 | 4.1 | 10.2 | 4.1 | 2.0 |  |
| Educational background (%) |  |  |  |  |  | 0.44 |  |  |  |  |  | 0.64 |
| Junior high school or other | 2.0 | 4.1 | 2.0 | 2.0 | 0.0 |  | 3.1 | 6.1 | 0.0 | 2.0 | 4.1 |  |
| Senior high school | 19.4 | 18.4 | 20.4 | 20.4 | 18.4 |  | 33.7 | 34.7 | 34.7 | 32.7 | 32.7 |  |
| Vocational school or junior college | 28.6 | 38.8 | 32.7 | 22.4 | 20.4 |  | 44.9 | 38.8 | 42.9 | 44.9 | 53.1 |  |
| University or graduate school | 50.0 | 38.8 | 44.9 | 55.1 | 61.2 |  | 18.4 | 20.4 | 22.4 | 20.4 | 10.2 |  |
| Smoking habit (%) |  |  |  |  |  | 0.78 |  |  |  |  |  | 0.34 |
| Nonsmoker | 33.7 | 34.7 | 38.8 | 26.5 | 34.7 |  | 78.6 | 73.5 | 77.6 | 85.7 | 77.6 |  |
| Past smoker | 29.1 | 26.5 | 24.5 | 30.6 | 34.7 |  | 7.1 | 4.1 | 6.1 | 8.2 | 10.2 |  |
| Current smoker | 37.2 | 38.8 | 36.7 | 42.9 | 30.6 |  | 14.3 | 22.4 | 16.3 | 6.1 | 12.2 |  |

GHGE, greenhouse gas emissions; CO_2_-eq, carbon dioxide equivalents.

*†Maen values within a row with different symbols were significantly different between the quartile group by post hoc Bonferroni’s test (P<0.05).

^a^ Participants (196 men and 196 women) were divided into quartiles by usual diet-related GHGE. Usual diet-related GHGE was calculated using the Multiple Source Method [43,44] and then adjusted for energy intake by residual method. Values are means ± SDs unless otherwise indicated.

^b^ Usual diet-related GHGE (g CO_2_-eq/d): median (25th, 75th percentiles).

^c^ Trend of association was examined for age and body mass index using a linear regression model with the median value of diet-related GHGE in each quartile as a continuous variable. χ2 test was used for categorical variables.

# S3 Table. Usual nutrient intake according to quartile (Q) of diet-related GHGE (g CO_2_-eq/day) among 196 Japanese men (aged 20-69 y) ^a^

|  | All (n=196) | | | Q1 (n=49) | Q2 (n=49) | Q3 (n=49) | Q4 (n=49) | P for trend^c^ |
| --- | --- | --- | --- | --- | --- | --- | --- | --- |
|  |  | Participants outside or below reference value (%) | | 3550 (3399, 3673) ^b^ | 4044 (3927, 4229) ^b^ | 4511 (4397, 4617) ^b^ | 5174 (4934, 5473) ^b^ |  |
| Energy (kcal/d) | 2348 ± 415 |  | | 2306 ± 476 | 2359 ± 409 | 2386 ± 376† | 2342 ± 400 | 0.81 |
| Nutrients with DG | | <DG | >DG |  |  |  |  |  |
| Protein (% energy) | 13.9 ± 1.4 | 27.0 |  | 12.8 ± 1.2* | 14.0 ± 1.2† | 14.0 ± 1.2† | 14.9 ± 1.2‡ | <.0001 |
| Total fat (% energy) | 26.7 ± 3.6 | 4.1 | 18.9 | 25.7 ± 3.7 | 27.5 ± 3.8 | 26.9 ± 3.5 | 26.9 ± 3.2 | 0.10 |
| Saturated fat (% energy) | 7.6 ± 1.4 | 65.8 |  | 6.8 ± 3.5 | 5.2 ± 1.7 | 6.5 ± 3.1 | 5.3 ± 1.9 | 0.23 |
| Carbohydrate (% energy) | 53.0 ± 6.1 | 27.6 | 1.5 | 56.2 ± 5.5* | 53.3 ± 6.1*† | 51.4 ± 6.3† | 51.2 ± 5.5† | <.0001 |
| Dietary fiber (g/d) | 14.1 ± 4.1 | 93.4 |  | 12.6 ± 3.8* | 14.5 ± 4.6*† | 14.0 ± 3.2*† | 15.4 ± 4.2† | 0.01 |
| Sodium (g NaCl equivalent/d) | 11.1 ± 2.3 | 92.9 |  | 10.4 ± 2.3* | 11.1 ± 2.2*† | 11.0 ± 1.9*† | 11.7 ± 2.8† | 0.06 |
| Potassium (mg/d) | 2710 ± 669 | 70.4 |  | 2284 ± 569* | 2711 ± 635† | 2821 ± 612† | 3023 ± 647† | <.0001 |
| Nutrient with the World Health Organization’s conditional recommendation^d^ | | ≥5% Energy | |  |  |  |  |  |
| Free sugar (% energy) | 6.3 ± 3.2 | 59.2 | | 7.3 ± 4.6* | 5.5 ± 1.8† | 6.8 ± 3.2*† | 5.5 ± 2.0† | 0.007 |
| Nutrients with EAR^e^ | | <EAR (%) | |  |  |  |  |  |
| Protein (g/d) | 81.0 ± 15.6 | 2.0 | | 72.4 ± 14.9* | 81.6 ± 14.9† | 83.1 ± 15.2† | 86.8 ± 14.2† | <.0001 |
| Vitamin A (μg RAE/d) | 539 ± 250 | 69.4 | | 401 ± 146* | 580 ± 291† | 573 ± 166† | 603 ± 304† | <.0001 |
| Thiamin (mg/d) | 1.1 ± 0.2 | 66.3 | | 1.0 ± 0.2* | 1.1 ± 0.2*† | 1.1 ± 0.3*† | 1.1 ± 0.2† | 0.003 |
| Riboflavin (mg/d) | 1.4 ± 0.3 | 38.8 | | 1.2 ± 0.3* | 1.4 ± 0.4† | 1.4 ± 0.3† | 1.5 ± 0.3† | <.0001 |
| Niacin (mg/d) | 20.5 ± 5.1 | 6.6 | | 17.6 ± 4.8* | 19.3 ± 3.8*† | 21.6 ± 5.1†‡ | 23.6 ± 4.6‡ | <.0001 |
| Vitamin B-6 (mg/d) | 1.4 ± 0.4 | 32.1 | | 1.2 ± 0.3* | 1.4 ± 0.3† | 1.4 ± 0.3†‡ | 1.6 ± 0.4‡ | <.0001 |
| Vitamin B-12 (μg/d) | 6.8 ± 2.8 | 0.5 | | 5.2 ± 2.2* | 7.2 ± 2.7† | 6.8 ± 2.7† | 8.0 ± 3.0† | <.0001 |
| Folate (μg/d) | 368 ± 122 | 5.6 | | 298 ± 103* | 376 ± 142† | 376 ± 106† | 423 ± 102† | <.0001 |
| Vitamin C (mg/d) | 107 ± 41 | 35.2 | | 82 ± 31* | 110 ± 41† | 112 ± 38† | 126 ± 40† | <.0001 |
| Calcium (mg/d) | 508 ± 153 | 71.9 | | 445 ± 150* | 546 ± 172† | 528 ± 128† | 515 ± 141*† | 0.006 |
| Magnesium (mg/d) | 299 ± 78 | 49.0 | | 266 ± 78* | 296 ± 76*† | 309 ± 81† | 324 ± 66† | 0.002 |
| Iron (mg/d)^d^ | 8.6 ± 2.0 | 12.8 | | 7.5 ± 1.9* | 8.6 ± 2.0† | 8.8 ± 1.8† | 9.5 ± 1.8† | <.0001 |
| Zinc (mg/d) | 9.4 ± 2.1 | 23.0 | | 8.5 ± 2.1* | 9.8 ± 2.4† | 9.3 ± 2.0*† | 10.1 ± 1.7† | 0.001 |
| Copper (mg/d) | 1.3 ± 0.3 | 2.6 | | 1.2 ± 0.3 | 1.3 ± 0.3 | 1.3 ± 0.3 | 1.3 ± 0.2 | 0.18 |

GHGE, greenhouse gas emission; CO_2_-eq, carbon dioxide equivalents; DG, Tentative Dietary Goal for Preventing Lifestyle-related Diseases; EAR, Estimated Average Requirement; RAE, retinol activity equivalent.

*†‡Maen values within a row with different symbols were significantly different between the quartile group by post hoc Bonferroni’s test (P<0.05).

^a^Participants were divided into quartiles by usual diet-related GHGE. Usual nutrient intake and diet-related GHGE were calculated using the Multiple Source Method [43,44]. Diet-related GHGE was adjusted for energy intake by residual method. Values are means ± SDs unless otherwise indicated.

^b^ Usual diet-related GHGE (g CO_2_-eq/d): median (25th, 75th percentiles).

^c^ Trend of association was examined using a linear regression model with the median value in each quartile as a continuous variable.

^d^ Probability approach was used to assess inadequacy for iron intake.

# S4 Table. Usual nutrient intake according to quartile (Q) of diet-related GHGE (g CO_2_-eq/day) among 196 Japanese women (aged 20-69 y) ^a^

|  | All (n=196) | | | Q1 (n=49) | Q2 (n=49) | Q3 (n=49) | Q4 (n=49) | P for trend^c^ |
| --- | --- | --- | --- | --- | --- | --- | --- | --- |
|  |  | Participants outside or below reference value (%) | | 3031 (2840, 3204)^b^ | 3450 (3405, 3587)^b^ | 3844 (3781, 3955)^b^ | 4420 (4270, 4574) ^b^ |  |
| Energy (kcal/d) | 1882 ± 310 |  | | 1916 ± 369 | 1859 ± 304 | 1798 ± 275 | 1955 ± 266 | 0.07 |
| Nutrients with DG | | <DG | >DG |  |  |  |  |  |
| Protein (% energy) | 14.3±1.4 | 17.3 | - | 13.6 ± 1.3* | 14.1 ± 1.2*† | 14.7 ± 1.6†‡ | 15.0 ± 1.3‡ | <.0001 |
| Total fat (% energy) | 29.0±3.6 | 0.5 | 37.2 | 29.2 ± 3.2 | 29.7 ± 4.3 | 28.2 ± 3.5 | 29.0 ± 3.4 | 0.20 |
| Saturated fat (% energy) | 8.6±1.6 | - | 84.7 | 8.6 ± 1.7 | 9.0 ± 1.8 | 8.4 ± 1.7 | 8.5 ± 1.3 | 0.28 |
| Carbohydrate (% energy) | 54.1±4.4 | 17.9 | 0.5 | 54.2 ± 4.3 | 53.7 ± 4.7 | 54.7 ± 4.6 | 53.8 ± 3.9 | 0.71 |
| Dietary fiber (g/d) | 13.7±4.0 | 89.3 | - | 12.1 ± 3.7* | 13.2 ± 3.4* | 13.8 ± 3.3*† | 15.8 ± 4.8† | <.0001 |
| Sodium (g NaCl equivalent/d) | 9.2±2.2 | - | 0.0 | 8.9 ± 2.1* | 9.0 ± 1.9* | 8.9 ± 1.8* | 10.1 ± 2.5† | 0.01 |
| Potassium (mg/d) | 2566±651 | 54.6 | - | 2301 ± 587* | 2446 ± 499* | 2497 ± 512* | 3018 ± 752† | <.0001 |
| Nutrient with the World Health Organization’s conditional recommendation^d^ | | ≥5% Energy | |  |  |  |  |  |
| Free sugar (% energy) | 7.5±2.7 | 84.2 | | 7.3 ± 3.1 | 7.2 ± 2.4 | 8.0 ± 2.8 | 7.6 ± 2.4 | 0.47 |
| Nutrients with EAR^e^ | | <EAR (%) | | <EAR (%) |  |  |  |  |
| Protein (g/d) | 67.0±12.4 | 1.0 | | 64.1 ± 12.9* | 65.2 ± 11.8* | 65.8 ± 10.6* | 72.9 ± 12.4† | 0.001 |
| Vitamin A (μg RAE/d) | 508 ± 168 | 52.0 | | 460 ± 140* | 470 ± 127* | 512 ± 150*† | 590 ± 215† | 0.0003 |
| Thiamin (mg/d) | 0.9±0.2 | 54.1 | | 0.9 ± 0.2 | 0.9 ± 0.2 | 0.9 ± 0.2 | 1.0 ± 0.2 | 0.04 |
| Riboflavin (mg/d) | 1.3±0.3 | 20.4 | | 1.2 ± 0.3* | 1.2 ± 0.3*† | 1.2 ± 0.3*† | 1.4 ± 0.3† | 0.006 |
| Niacin (mg/d) | 16.8±3.6 | 2.0 | | 15.0 ± 3.5* | 16.3 ± 3.2*† | 17.2 ± 3.5†‡ | 18.7 ± 3.4‡ | <.0001 |
| Vitamin B-6 (mg/d) | 1.2±0.3 | 24.5 | | 1.1 ± 0.3* | 1.1 ± 0.3* | 1.2 ± 0.2* | 1.3 ± 0.3† | <.0001 |
| Vitamin B-12 (μg/d) | 5.8±2.2 | 0.0 | | 5.1 ± 1.9* | 5.4 ± 1.7*† | 6.2 ± 2.6*† | 6.4 ± 2.3† | 0.01 |
| Folate (μg/d) | 366±127 | 6.6 | | 310 ± 110* | 338 ± 91† | 372 ± 124† | 443 ± 141† | <.0001 |
| Vitamin C (mg/d) | 116±48 | 27.0 | | 99 ± 41* | 105 ± 35* | 114 ± 44* | 144 ± 58† | <.0001 |
| Calcium (mg/d) | 510±158 | 64.8 | | 489 ± 164* | 496 ± 132* | 476 ± 141* | 580 ± 175† | 0.004 |
| Magnesium (mg/d) | 275±71 | 28.6 | | 251 ± 75* | 267 ± 54* | 267 ± 53* | 315 ± 81† | <.0001 |
| Iron (mg/d)^d^ | 7.9±2.0 | 54.6 | | 7.2 ± 2.1* | 7.6 ± 1.7* | 7.9 ± 1.6* | 8.9 ± 2.3† | 0.0002 |
| Zinc (mg/d) | 7.7±1.5 | 11.2 | | 7.4 ± 1.4* | 7.4 ± 1.4* | 7.7 ± 1.5* | 8.5 ± 1.6† | 0.0002 |
| Copper (mg/d) | 1.1±0.3 | 1.0 | | 1.1 ± 0.3* | 1.1 ± 0.2* | 1.1 ± 0.2* | 1.2 ± 0.3† | 0.002 |

GHGE, greenhouse gas emission; CO_2_-eq, carbon dioxide equivalents; DG, Tentative Dietary Goal for Preventing Lifestyle-related Diseases; EAR, Estimated Average Requirement; RAE, retinol activity equivalent.

*†‡Maen values within a row with different symbols were significantly different between the quartile group by post hoc Bonferroni’s test (P<0.05).

^a^Participants were divided into quartiles by usual diet-related GHGE. Usual nutrient intake and diet-related GHGE were calculated using the Multiple Source Method [43,44]. Diet-related GHGE was adjusted for energy intake by residual method. Values are means ± SDs unless otherwise indicated.

^b^ Usual diet-related GHGE (g CO_2_-eq/d): median (25th, 75th percentiles).

^c^ Trend of association was examined using a linear regression model with the median value in each quartile as a continuous variable.

^d^ Probability approach was used to assess inadequacy for iron intake.

# S5 Table. Odds ratios for inadequate nutrient intake compared to DRI reference value according to the quartile (Q) of usual diet-related GHGE (g CO_2_-eq/day) among 196 Japanese men and 196 women (aged 20-69 y)^a^

|  | Diet-related GHGE | Men (n=196) | | Women (n=196) | |
| --- | --- | --- | --- | --- | --- |
|  | Diet-related GHGE | Inadequate/adequate intake participants (n)^b^ | OR (95% CI) | Inadequate/adequate intake participants (n)^b^ | OR (95% CI) |
| Nutrients with Tentative Dietary Goal for Preventing Lifestyle-related Diseases | | | |  |  |
| Protein | Q1 | 31/18 | 1 (ref) | 16/33 | 1 (ref) |
|  | Q2 | 9/40 | 0.13 (0.05-0.33) | 9/40 | 0.46 (0.18-1.19) |
|  | Q3 | 10/39 | 0.15 (0.06-0.37) | 6/43 | 0.29 (0.10-0.82) |
|  | Q4 | 3/46 | 0.04 (0.01-0.14) | 3/46 | 0.14 (0.04-0.50) |
|  |  |  | P for trend^c^ <.0001 |  | P for trend^c^=0.0009 |
| Total fat | Q1 | 11/38 | 1 (ref) | 19/30 | 1 (ref) |
|  | Q2 | 15/34 | 1.52 (0.62-3.77) | 23/26 | 1.40 (0.63-3.12) |
|  | Q3 | 10/39 | 0.89 (0.34-2.33) | 13/36 | 0.57 (0.24-1.34) |
|  | Q4 | 9/40 | 0.78 (0.29-2.09) | 19/30 | 1.00 (0.44-2.25) |
|  |  |  | P for trend^c^=0.40 |  | P for trend^c^=0.59 |
| Saturated fat | Q1 | 28/21 | 1 (ref) | 43/6 | 1 (ref) |
|  | Q2 | 33/16 | 1.55 (0.68-3.52) | 42/7 | 0.84 (0.26-2.70) |
|  | Q3 | 34/15 | 1.70 (0.74-3.90) | 39/10 | 0.54 (0.18-1.64) |
|  | Q4 | 34/15 | 1.70 (0.74-3.90) | 42/7 | 0.84 (0.26-2.70) |
|  |  |  | P for trend^c^=0.22 |  | P for trend^c^=0.66 |
| Carbohydrate | Q1 | 11/38 | 1 (ref) | 10/39 | 1 (ref) |
|  | Q2 | 13/36 | 1.25 (0.50-3.14) | 11/38 | 1.13 (0.43-2.97) |
|  | Q3 | 15/34 | 1.52 (0.62-3.77) | 8/41 | 0.76 (0.27-2.13) |
|  | Q4 | 18/31 | 2.01 (0.83-4.87) | 7/42 | 0.65 (0.23-1.88) |
|  |  |  | P for trend^c^=0.11 |  | P for trend^c^=0.32 |
| Dietary fibre | Q1 | 47/2 | 1 (ref) | 46/3 | 1 (ref) |
|  | Q2 | 46/3 | 0.65 (0.10-4.09) | 44/5 | 0.57 (0.13-2.55) |
|  | Q3 | 46/3 | 0.65 (0.10-4.09) | 46/3 | 1.00 (0.19-5.22) |
|  | Q4 | 44/5 | 0.37 (0.07-2.03) | 39/10 | 0.25 (0.07-0.99) |
|  |  |  | P for trend^c^=0.24 |  | P for trend^c^=0.04 |
| Sodium | Q1 | 39/10 | 1 (ref) | 42/7 | 1 (ref) |
|  | Q2 | 47/2 | 6.03 (1.25-29.15) | 42/7 | 1.00 (0.32-3.10) |
|  | Q3 | 47/2 | 6.03 (1.25-29.15) | 42/7 | 1.00 (0.32-3.10) |
|  | Q4 | 49/0 | - | 47/2 | 3.92 (0.77-19.90) |
|  |  |  | P for trend^c^=0.002 |  | P for trend^c^=0.12 |
| Potassium | Q1 | 44/5 | 1 (ref) | 34/15 | 1 (ref) |
|  | Q2 | 35/14 | 0.28 (0.09-0.87) | 30/19 | 0.70 (0.30-1.61) |
|  | Q3 | 35/14 | 0.28 (0.09-0.87) | 30/19 | 0.70 (0.30-1.61) |
|  | Q4 | 24/25 | 0.11 (0.04-0.32) | 13/36 | 0.16 (0.07-0.38) |
|  |  |  | P for trend^c^ <.0001 |  | P for trend^c^<.0001 |
| Nutrient with the World Health Organization’s conditional recommendation^e^ | | | |  |  |
| Free sugar | Q1 | 32/17 | 1 (ref) | 37/12 | 1 (ref) |
|  | Q2 | 27/22 | 0.65 (0.29-1.47) | 43/6 | 2.32 (0.79-6.80) |
|  | Q3 | 32/17 | 1.00 (0.44-2.30) | 41/8 | 1.66 (0.61-4.51) |
|  | Q4 | 25/24 | 0.55 (0.25-1.25) | 44/5 | 2.85 (0.92-8.85) |
|  |  |  | P for trend^c^=0.27 |  | P for trend^c^=0.10 |
| Nutrients with Estimated Average Requirement^c^ | | | |  |  |
| Vitamin A | Q1 | 44/5 | 1 (ref) | 31/18 | 1 (ref) |
|  | Q2 | 29/20 | 0.17 (0.06-0.49) | 27/22 | 0.71 (0.32-1.60) |
|  | Q3 | 33/16 | 0.23 (0.08-0.71) | 28/21 | 0.77 (0.34-1.74) |
|  | Q4 | 30/19 | 0.18 (0.06-0.53) | 16/33 | 0.28 (0.12-0.65) |
|  |  |  | P for trend^c^=0.01 |  | P for trend^c^=0.004 |
| Thiamine | Q1 | 39/10 | 1 (ref) | 32/17 | 1 (ref) |
|  | Q2 | 36/13 | 0.71 (0.28-1.82) | 25/24 | 0.55 (0.25-1.25) |
|  | Q3 | 29/20 | 0.37 (0.15-0.91) | 31/18 | 0.92 (0.40-2.09) |
|  | Q4 | 26/23 | 0.29 (0.12-0.71) | 18/31 | 0.31 (0.14-0.71) |
|  |  |  | P for trend^c^=0.0025 |  | P for trend^c^=0.01 |
| Riboflavin | Q1 | 29/20 | 1 (ref) | 19/30 | 1 (ref) |
|  | Q2 | 20/29 | 0.48 (0.21-1.07) | 9/40 | 0.36 (0.14-0.90) |
|  | Q3 | 17/32 | 0.37 (0.16-0.83) | 7/42 | 0.26 (0.10-0.71) |
|  | Q4 | 10/39 | 0.18 (0.07-0.43) | 5/44 | 0.18 (0.06-0.53) |
|  |  |  | P for trend^c^ =0.0001 |  | P for trend^c^=0.001 |
| Vitamin B-6 | Q1 | 30/19 | 1 (ref) | 22/27 | 1 (ref) |
|  | Q2 | 18/31 | 0.37 (0.16-0.83) | 11/38 | 0.36 (0.15-0.85) |
|  | Q3 | 10/39 | 0.16 (0.07-0.40) | 10/39 | 0.32 (0.13-0.77) |
|  | Q4 | 5/44 | 0.07 (0.02-0.21) | 5/44 | 0.14 (0.05-0.41) |
|  |  |  | P for trend^c^<.0001 |  | P for trend^c^=0.0002 |
| Vitamin C | Q1 | 29/20 | 1 (ref) | 21/28 | 1 (ref) |
|  | Q2 | 16/33 | 0.33 (0.15-0.76) | 16/33 | 0.65 (0.28-1.47) |
|  | Q3 | 15/34 | 0.30 (0.13-0.70) | 13/36 | 0.48 (0.21-1.13) |
|  | Q4 | 9/40 | 0.16 (0.06-0.39) | 3/46 | 0.09 (0.02-0.32) |
|  |  |  | P for trend^c^ <.0001 |  | P for trend^c^<.0001 |
| Calcium | Q1 | 40/9 | 1 (ref) | 32/17 | 1 (ref) |
|  | Q2 | 33/16 | 0.46 (0.18-1.19) | 36/13 | 1.47 (0.62-3.49) |
|  | Q3 | 33/16 | 0.46 (0.18-1.19) | 36/13 | 1.47 (0.62-3.49) |
|  | Q4 | 35/14 | 0.56 (0.22-1.46) | 23/26 | 0.47 (0.21-1.06) |
|  |  |  | P for trend^c^ =0.33 |  | P for trend^c^=0.04 |
| Magnesium | Q1 | 34/15 | 1 (ref) | 22/27 | 1 (ref) |
|  | Q2 | 22/27 | 0.36 (0.16-0.82) | 14/35 | 0.49 (0.21-1.13) |
|  | Q3 | 25/24 | 0.46 (0.20-1.05) | 13/36 | 0.44 (0.19-1.04) |
|  | Q4 | 15/34 | 0.20 (0.08-0.46) | 7/42 | 0.21 (0.08-0.54) |
|  |  |  | P for trend^c^=0.0006 |  | P for trend^c^=0.002 |
| Iron^d^ | Q1 | 13/36 | 1 (ref) | 33/16 | 1 (ref) |
|  | Q2 | 7/42 | 0.46 (0.17-1.28) | 26/23 | 0.55 (0.24-1.24) |
|  | Q3 | 2/47 | 0.12 (0.03-0.56) | 30/19 | 0.77 (0.33-1.75) |
|  | Q4 | 3/46 | 0.18 (0.05-0.68) | 18/31 | 0.28 (0.12-0.65) |
|  |  |  | P for trend^c^=0.002 |  | P for trend^c^=0.007 |
| Zinc | Q1 | 18/31 | 1 (ref) | 11/38 | 1 (ref) |
|  | Q2 | 11/38 | 0.50 (0.21-1.21) | 6/43 | 0.48 (0.16-1.43) |
|  | Q3 | 11/38 | 0.50 (0.21-1.21) | 4/45 | 0.31 (0.09-1.04) |
|  | Q4 | 5/44 | 0.20 (0.07-0.58) | 1/48 | 0.07 (0.01-0.58) |
|  |  |  | P for trend^c^=0.004 |  | P for trend^c^=0.003 |

GHGE, greenhouse gas emissions; CO_2_-eq, carbon dioxide equivalents.

^a^ Participants were divided into quartiles by usual diet-related GHGE. Usual food intake and diet-related GHGE were calculated using the Multiple Source Method [43,44]. Diet-related GHGE was adjusted for energy intake by residual method.

^b^ Usual diet-related GHGE (g CO_2_-eq/day): median (25th-75th percentiles).

^c^ Trend of association was examined using a linear regression model with the median value in each quartile as a continuous variable.

# S6 Table**.** Usual food intake (g/d) according to quartile (Q) of usual diet-related GHGE (g CO_2_-eq/day) among 196 Japanese men and 196 women (aged 20-69 y)^a^

|  | Men | | | | | | Women | | | | | |
| --- | --- | --- | --- | --- | --- | --- | --- | --- | --- | --- | --- | --- |
|  | All (n=196) | Q1 (n=98) | Q2 (n=98) | Q3 (n=98) | Q4 (n=98) | P for trend^c^ | All (n=196) | Q1 (n=98) | Q2 (n=98) | Q3 (n=98) | Q4 (n=98) | P for trend^c^ |
| Cereals | 523 ± 131 | 563 ± 166* | 542 ± 130*† | 492 ± 114† | 497 ± 93*† | 0.015 | 374 ± 83 | 400 ± 85 | 375 ± 79 | 357 ± 91 | 364 ± 70 | 0.06 |
| Potatoes | 48 ± 17 | 45 ± 17 | 49 ± 16 | 46 ± 14 | 53 ± 21 | 0.17 | 42 ± 16 | 39 ± 15* | 41 ± 14* | 37 ± 13* | 52 ± 18† | <.0001 |
| Sugar | 15 ± 10 | 16 ± 12 | 15 ± 9 | 15 ± 9 | 15 ± 10 | 0.84 | 15 ± 9 | 14 ± 9 | 14 ± 7 | 18 ± 10 | 16 ± 9 | 0.05 |
| Pulses | 56 ± 35 | 50 ± 35 | 55 ± 34 | 57 ± 39 | 60 ± 31 | 0.52 | 56 ± 34 | 49 ± 32 | 58 ± 30 | 54 ± 27 | 64 ± 43 | 0.14 |
| Nuts | 3 ± 6 | 3 ± 7 | 4 ± 5 | 4 ± 7 | 2 ± 3 | 0.53 | 3 ± 3 | 3 ± 3 | 2 ± 2 | 3 ± 3 | 4 ± 4 | 0.17 |
| Vegetables | 246 ± 89 | 185 ± 63* | 251 ± 88† | 258 ± 73† | 291 ± 97† | <.0001 | 245 ± 97 | 206 ± 80* | 224 ± 68* | 247 ± 85* | 301 ± 123† | <.0001 |
| Fruits | 79 ± 74 | 60 ± 74 | 91 ± 81 | 79 ± 64 | 85 ± 73 | 0.17 | 92 ± 80 | 88 ± 83 | 78 ± 58 | 90 ± 71 | 114 ± 99 | 0.15 |
| Mushroom | 14 ± 9 | 12 ± 9* | 14 ± 8*† | 14 ± 8*† | 17 ± 11† | 0.05 | 16 ± 11 | 12 ± 8* | 14 ± 10*† | 19 ± 13† | 19 ± 12† | 0.00 |
| Seaweeds | 5 ± 4 | 5 ± 4 | 5 ± 3 | 5 ± 4 | 6 ± 5 | 0.21 | 5 ± 4 | 4 ± 3* | 5 ± 4*† | 6 ± 4† | 6 ± 4*† | 0.02 |
| Fish and seafood | 45 ± 22 | 33 ± 17* | 46 ± 23† | 49 ± 23† | 52 ± 22† | <.0001 | 36 ± 18 | 29 ± 15* | 35 ± 15*† | 36 ± 19*† | 42 ± 21† | 0.01 |
| Meat | 109 ± 42 | 92 ± 36* | 104 ± 41*† | 113 ± 44*† | 125 ± 41† | 0.001 | 79 ± 29 | 79 ± 29 | 77 ± 31 | 81 ± 28 | 80 ± 29 | 0.91 |
| Beef | 19 ± 12 | 13 ± 8* | 17 ± 11*† | 20 ± 11† | 27 ± 15‡ | <.0001 | 13 ± 9 | 9 ± 5* | 9 ± 6* | 14 ± 8† | 19 ± 11‡ | <.0001 |
| Pork | 40 ± 17 | 37 ± 17 | 38 ± 16 | 41 ± 19 | 45 ± 17 | 0.10 | 29 ± 14 | 30 ± 14 | 31 ± 17 | 27 ± 12 | 26 ± 12 | 0.24 |
| Chicken | 34 ± 16 | 33 ± 15 | 33 ± 16 | 37 ± 19 | 35 ± 15 | 0.53 | 27 ± 13 | 29 ± 11 | 25 ± 14 | 29 ± 14 | 26 ± 13 | 0.19 |
| Processed meat products | 14 ± 9 | 12 ± 8 | 15 ± 10 | 16 ± 12 | 12 ± 7 | 0.10 | 11 ± 7 | 12 ± 7 | 11 ± 8 | 10 ± 7 | 10 ± 7 | 0.46 |
| Egg | 43 ± 14 | 41 ± 14 | 44 ± 16 | 43 ± 15 | 43 ± 12 | 0.80 | 36 ± 13 | 39 ± 14 | 34 ± 15 | 35 ± 12 | 38 ± 12 | 0.19 |
| Milk and dairy food products | 90 ± 82 | 74 ± 73* | 122 ± 101† | 85 ± 61*† | 78 ± 79* | 0.01 | 106 ± 81 | 111 ± 89 | 112 ± 85 | 87 ± 67 | 116 ± 82 | 0.29 |
| Fat and oils | 22 ± 7 | 23 ± 7* | 24 ± 7* | 23 ± 7*† | 19 ± 6† | 0.001 | 20 ± 6 | 22 ± 6* | 20 ± 6*† | 17 ± 5† | 19 ± 5*† | 0.0027 |
| Confectioneries | 38 ± 28 | 39 ± 34 | 35 ± 21 | 44 ± 31 | 32 ± 22 | 0.14 | 46 ± 25 | 47 ± 28 | 46 ± 24 | 45 ± 27 | 48 ± 20 | 0.95 |
| Alcohol beverages | 195 ± 280 | 178 ± 277 | 128 ± 183 | 249 ± 265 | 227 ± 361 | 0.14 | 68 ± 117 | 69 ± 110 | 50 ± 70 | 82 ± 153 | 72 ± 122 | 0.62 |
| Tea and coffee | 584 ± 361 | 511 ± 292 | 561 ± 360 | 590 ± 412 | 675 ± 363 | 0.15 | 613 ± 350 | 509 ± 327* | 560 ± 325* | 634 ± 306*† | 750 ± 396† | 0.004 |
| Sweetened beverages | 45 ± 87 | 75 ± 121* | 34 ± 47*† | 50 ± 103*† | 23 ± 42† | 0.02 | 32 ± 55 | 40 ± 64 | 35 ± 69 | 29 ± 40 | 25 ± 40 | 0.53 |
| Seasonings | 126 ± 72 | 87 ± 37* | 113 ± 50*† | 126 ± 61† | 177 ± 96‡ | <.0001 | 113 ± 63 | 80 ± 31* | 99 ± 49*† | 115 ± 52† | 158 ± 81‡ | <.0001 |
| Water | 527 ± 353 | 460 ± 287 | 510 ± 309 | 644 ± 444 | 493 ± 334 | 0.05 | 504 ± 295 | 451 ± 246 | 512 ± 264 | 483 ± 268 | 570 ± 378 | 0.23 |

GHGE, greenhouse gas emissions; CO_2_-eq, carbon dioxide equivalents.

*†‡Maen values within a row with different symbols were significantly different between the quartile group by post hoc Bonferroni’s test (P<0.05).

^a^ Participants were divided into quartiles by usual diet-related GHGE separately by sex. Usual food intake and diet-related GHGE were calculated using the Multiple Source Method [43,44]. Diet-related GHGE was adjusted for energy intake by residual method. Values are means ± SDs.

^b^ Usual diet-related GHGE (g CO_2_-eq/d): median (25th-75th percentiles).

^c^ Trend of association was examined using a linear regression model with the median value in each quartile as a continuous variable.

# S7 Table. Basic characteristics of participants according to quartile (Q) of diet-related GHGE (g CO_2_-eq/day) among 369 Japanese adults with plausible energy intake^a^

|  | All (n=369) | Q1 (n=92) | Q2 (n=92) | Q3 (n=93) | Q4 (n=92) | P^c^ |
| --- | --- | --- | --- | --- | --- | --- |
|  |  | 3242 (3000, 3539)^b^ | 3786 (3469, 4077)^b^ | 4074 (3843, 4524)^b^ | 4822 (4398, 5198)^b^ |  |
| Age (years) | 44.7 ± 13.2 | 39.8 ± 13.1* | 44.7 ± 13.2*† | 44.8 ± 13.3*† | 49.6 ± 11.7† | <.0001 |
| Body mass index (kg/m^2^) | 23.2 ± 3.4 | 23.0 ± 3.7 | 23.5 ± 3.9 | 22.8 ± 2.9 | 23.5 ± 3.2 | 0.36 |
| Living area (%) |  |  |  |  |  | 0.02 |
| Hokkaido and Tohoku | 14.9 | 16.3 | 13.0 | 7.5 | 13.0 |  |
| Kanto | 19.8 | 23.9 | 14.1 | 23.7 | 14.1 |  |
| Hokuriku and Tokai | 10.0 | 10.9 | 5.4 | 12.9 | 5.4 |  |
| Kinki | 15.4 | 7.6 | 23.9 | 17.2 | 23.9 |  |
| Chugoku and Shikoku | 20.1 | 26.1 | 17.4 | 20.4 | 17.4 |  |
| Kyusyu and Okinawa | 19.8 | 15.2 | 26.1 | 20.4 | 27.2 |  |
| Occupation (%) |  |  |  |  |  | 0.004 |
| Clerical | 43.6 | 42.4 | 43.5 | 51.6 | 44.6 |  |
| Nursing care | 40.7 | 42.4 | 39.1 | 33.3 | 39.1 |  |
| Medical assistant | 3.3 | 0.0 | 1.1 | 9.7 | 1.1 |  |
| Cooking assistant | 5.7 | 5.4 | 9.8 | 3.2 | 9.8 |  |
| Others | 6.8 | 9.8 | 6.5 | 4.3 | 6.5 |  |
| Educational background (%) |  |  |  |  |  | 0.83 |
| Junior high school or other | 2.7 | 1.1 | 2.2 | 2.2 | 2.2 |  |
| Senior high school | 25.7 | 25.0 | 26.1 | 28.0 | 26.1 |  |
| Vocational school or junior college | 36.0 | 39.1 | 34.8 | 34.4 | 34.8 |  |
| University or graduate school | 35.5 | 34.8 | 37.0 | 37.6 | 38.0 |  |
| Smoking habit (%) |  |  |  |  |  | 0.65 |
| Nonsmoker | 56.1 | 58.7 | 55.4 | 55.9 | 56.5 |  |
| Past smoker | 18.2 | 13.0 | 22.8 | 20.4 | 22.8 |  |
| Current smoker | 25.7 | 28.3 | 21.7 | 25.8 | 21.7 |  |

BMR, basal metabolic rate; EI, energy intake; GHGE greenhouse gas emissions; CO_2_-eq, carbon dioxide equivalents.

*†‡Maen values within a row with different symbols were significantly different between the quartile group by post hoc Bonferroni’s test (P<0.05).

^a^ Participants with plausible energy intake was defined as participants with an EI:BMR 1.02-2.35 for men and 1.03-2.36 for women. Participants (184 men and 185 women) were divided into quartiles by usual diet-related GHGE separately by sex, and then combined for analysis. Usual diet-related GHGE was calculated using the Multiple Source Method [43,44] and then adjusted for energy intake by residual method. Values are means ± SDs unless otherwise indicated.

^b^ Usual diet-related GHGE (g CO_2_-eq/d): median (25th, 75th percentiles).

^c^ Trend of association was examined for age and body

mass index using a linear regression model with the median value of diet-related GHGE in each quartile as a continuous variable. χ2 test was used for categorical variables.

# S8 Table. Usual nutrient intake according to quartile (Q) of diet-related GHGE (g CO_2_-eq/day) among 369 Japanese adults (aged 20-69 y) with plausible energy intake^a^

|  | All (n=369) | | | Q1 (n=92) | Q2 (n=92) | Q3 (n=93) | Q4 (n=92) | P for trend^c^ |
| --- | --- | --- | --- | --- | --- | --- | --- | --- |
|  |  | Participants outside or below reference value (%) | | 3242 (3000, 3539)^b^ | 3786 (3469, 4077)^b^ | 4074 (3843, 4524)^b^ | 4822 (4398, 5198)^b^ |  |
| Energy (kcal/d) | 2126 ± 407 |  | | 2103 ± 413 | 2129 ± 423 | 2118 ± 421 | 2154 ± 373 | 0.85 |
| Nutrients with DG | | <DG | >DG |  |  |  |  |  |
| Protein (% energy) | 14.2 ± 1.4 | 20.9 | - | 13.3 ± 1.3 | 14.0 ± 1.2*† | 14.4 ± 1.5*† | 14.9 ± 1.2† | <.0001 |
| Total fat (% energy) | 27.9 ± 3.8 | 2.2 | 29.0 | 27.6 ± 3.9 | 28.6 ± 4.2 | 27.5 ± 3.6 | 28.1 ± 3.4 | 0.18 |
| Saturated fat (% energy) | 8.1 ± 1.6 | - | 75.1 | 8.0 ± 1.8 | 8.4 ± 1.7 | 8.0 ± 1.6 | 8.1 ± 1.3 | 0.29 |
| Carbohydrate (% energy) | 53.5 ± 5.3 | 22.8 | 0.8 | 55.1 ± 4.9* | 53.4 ± 5.5*† | 53.1 ± 5.8† | 52.4 ± 4.8† | 0.004 |
| Dietary fiber (g/d) | 14.0 ± 3.9 | 92.1 | - | 12.4 ± 3.6* | 13.8 ± 3.6*† | 14.3 ± 3.5†‡ | 15.6 ± 4.1‡ | <.0001 |
| Sodium (g NaCl equivalent/d) | 10.2 ± 2.3 | - | 93.0 | 9.7 ± 2.2* | 10.1 ± 2.3*† | 10.2 ± 2.1*† | 10.9 ± 2.6† | 0.01 |
| Potassium (mg/d) | 2661 ± 623 | 62.1 | - | 2297 ± 505* | 2602 ± 534† | 2715 ± 607† | 3029 ± 616‡ | <.0001 |
| Nutrient with the World Health Organization’s conditional recommendation^d^ | | ≥5% Energy | |  |  |  |  |  |
| Free sugar (% energy) | 6.9 ± 3.0 | 71.8 | | 7.1 ± 3.8 | 6.4 ± 2.3 | 7.4 ± 3.1 | 6.5 ± 2.3 | 0.07 |
| Nutrients with EAR^e^ | | <EAR (%) | |  |  |  |  |  |
| Protein (g/d) | 74.6 ± 15.1 | 0.5 | | 68.5 ± 13.4* | 74.1 ± 15.4*† | 75.4 ± 15.2†‡ | 80.3 ± 14.3‡ | <.0001 |
| Vitamin A (μg RAE/d) | 528 ± 212 | 60.7 | | 425 ± 120* | 536 ± 230† | 548 ± 160† | 604 ± 268† | 0.0004 |
| Thiamin (mg/d) | 1.0 ± 0.2 | 59.6 | | 0.9 ± 0.2* | 1.0 ± 0.2*† | 1.0 ± 0.2*† | 1.1 ± 0.2† | <.0001 |
| Riboflavin (mg/d) | 1.3 ± 0.3 | 27.9 | | 1.2 ± 0.3* | 1.3 ± 0.3† | 1.3 ± 0.3† | 1.5 ± 0.3† | <.0001 |
| Niacin (mg/d) | 18.8 ± 4.6 | 3.0 | | 16.4 ± 4.0* | 18.0 ± 3.7*† | 19.6 ± 4.9†‡ | 21.2 ± 4.4‡ | <.0001 |
| Vitamin B-6 (mg/d) | 1.3 ± 0.3 | 26.3 | | 1.1 ± 0.3* | 1.2 ± 0.3† | 1.3 ± 0.3† | 1.5 ± 0.3‡ | <.0001 |
| Vitamin B-12 (μg/d) | 6.3 ± 2.6 | 0.3 | | 5.1 ± 1.8* | 6.4 ± 2.5† | 6.6 ± 2.6† | 7.2 ± 2.8† | <.0001 |
| Folate (μg/d) | 369 ± 119 | 4.9 | | 298 ± 90* | 362 ± 116† | 382 ± 116† | 433 ± 114‡ | <.0001 |
| Vitamin C (mg/d) | 112 ± 43 | 29.5 | | 89 ± 31* | 109 ± 37† | 115 ± 42† | 135 ± 48‡ | <.0001 |
| Calcium (mg/d) | 514 ± 149 | 68.0 | | 467 ± 153* | 530 ± 147† | 510 ± 135*† | 549 ± 152† | 0.002 |
| Magnesium (mg/d) | 289 ± 71 | 37.7 | | 260 ± 68* | 284 ± 65*† | 294 ± 71†‡ | 319 ± 66‡ | <.0001 |
| Iron (mg/d)^d^ | 8.3 ± 1.9 | 32.2 | | 7.3 ± 1.7* | 8.2 ± 1.8† | 8.5 ± 1.8† | 9.2 ± 1.8‡ | <.0001 |
| Zinc (mg/d) | 8.6 ± 2.0 | 14.6 | | 8.0 ± 1.7* | 8.7 ± 2.3*† | 8.6 ± 1.9*† | 9.3 ± 1.8† | <.0001 |
| Copper (mg/d) | 1.2 ± 0.3 | 0.0 | | 1.1 ± 0.3* | 1.2 ± 0.3*† | 1.2 ± 0.3*† | 1.3 ± 0.2† | 0.003 |

BMR, basal metabolic rate; EI, energy intake; GHGE, greenhouse gas emission; CO_2_-eq, carbon dioxide equivalents; DG, Tentative Dietary Goal for Preventing Lifestyle-related Diseases; EAR, Estimated Average Requirement; RAE, retinol activity equivalent.

*†‡Maen values within a row with different symbols were significantly different between the quartile group by post hoc Bonferroni’s test (P<0.05).

^a^ Participants with plausible energy intake was defined as participants with an EI:BMR 1.02-2.35 for men and 1.03-2.36 for women. Participants (184 men and 185 women) were divided into quartiles by usual diet-related GHGE separately by sex, and then combined for analysis. Usual diet-related GHGE was calculated using the Multiple Source Method [43,44] and then adjusted for energy intake by residual method. Values are means ± SDs unless otherwise indicated.

^b^ Usual diet-related GHGE (g CO_2_-eq/d): median (25th, 75th percentiles).

^c^ Trend of association was examined using a linear regression model with the median value in each quartile as a continuous variable.

^d^ Probability approach was used to assess inadequacy for iron intake.

# S9 Table. Odds ratios for inadequate nutrient intake compared to DRIs reference value according to the quartile (Q) of usual diet-related GHGE (g CO_2_-eq/day) among 369 Japanese adults (aged 20-69 y) with plausible energy reporting^a^

|  | Diet-related GHGE | Inadequate/adequate intake participants (n)^b^ | OR (95% CI) |
| --- | --- | --- | --- |
| Nutrients with Tentative Dietary Goal for Preventing Lifestyle-related Diseases | | | |
| Protein | Q1 | 40/52 | 1 (ref) |
|  | Q2 | 17/75 | 0.28 (0.14-0.55) |
|  | Q3 | 15/78 | 0.25 (0.13-0.50) |
|  | Q4 | 5/87 | 0.07 (0.03-0.19) |
|  |  |  | P for trend^c^<.0001 |
| Total fat | Q1 | 31/61 | 1 (ref) |
|  | Q2 | 35/57 | 1.29 (0.70-2.36) |
|  | Q3 | 21/72 | 0.61 (0.32-1.18) |
|  | Q4 | 28/64 | 0.88 (0.47-1.64) |
|  |  |  | P for trend^c^=0.28 |
| Saturated fat | Q1 | 67/25 | 1 (ref) |
|  | Q2 | 70/22 | 1.36 (0.70-2.66) |
|  | Q3 | 68/25 | 1.11 (0.58-2.13) |
|  | Q4 | 72/20 | 1.36 (0.70-2.66) |
|  |  |  | P for trend^c^=0.49 |
| Carbohydrate | Q1 | 18/74 | 1 (ref) |
|  | Q2 | 24/68 | 1.28 (0.63-2.57) |
|  | Q3 | 21/72 | 1.17 (0.58-2.37) |
|  | Q4 | 24/68 | 1.35 (0.68-2.71) |
|  |  |  | P for trend^c^=0.46 |
| Dietary fibre | Q1 | 88/4 | 1 (ref) |
|  | Q2 | 87/5 | 0.51 (0.12-2.11) |
|  | Q3 | 86/7 | 0.64 (0.15-2.74) |
|  | Q4 | 79/13 | 0.20 (0.06-0.72) |
|  |  |  | P for trend^c^=0.01 |
| Sodium | Q1 | 80/12 | 1 (ref) |
|  | Q2 | 85/7 | 1.06 (0.36-3.16) |
|  | Q3 | 87/6 | 1.97 (0.56-6.96) |
|  | Q4 | 91/1 | 7.95 (0.96-65.97) |
|  |  |  | P for trend^c^=0.03 |
| Potassium | Q1 | 75/17 | 1 (ref) |
|  | Q2 | 62/30 | 0.44 (0.22-0.89) |
|  | Q3 | 59/34 | 0.42 (0.21-0.83) |
|  | Q4 | 33/59 | 0.12 (0.06-0.24) |
|  |  |  | P for trend^c^ <.0001 |
| Nutrient with the World Health Organization’s conditional recommendation^e^ | | | |
| Free sugar | Q1 | 64/28 | 1 (ref) |
|  | Q2 | 66/26 | 1.14 (0.60-2.16) |
|  | Q3 | 69/24 | 1.24 (0.65-2.36) |
|  | Q4 | 66/26 | 1.14 (0.60-2.16) |
|  |  |  | P for trend^c^=0.64 |
| Nutrients with Estimated Average Requirement^c^ | | | |
| Vitamin A | Q1 | 73/19 | 1 (ref) |
|  | Q2 | 52/40 | 0.37 (0.19-0.70) |
|  | Q3 | 56/37 | 0.45 (0.23-0.87) |
|  | Q4 | 43/49 | 0.27 (0.14-0.52) |
|  |  |  | P for trend^c^=0.0003 |
| Thiamine | Q1 | 69/23 | 1 (ref) |
|  | Q2 | 57/35 | 0.59 (0.31-1.11) |
|  | Q3 | 54/39 | 0.53 (0.28-1.00) |
|  | Q4 | 40/52 | 0.27 (0.14-0.50) |
|  |  |  | P for trend^c^<.0001 |
| Riboflavin | Q1 | 47/45 | 1 (ref) |
|  | Q2 | 24/68 | 0.41 (0.22-0.75) |
|  | Q3 | 20/73 | 0.32 (0.17-0.60) |
|  | Q4 | 12/80 | 0.16 (0.07-0.32) |
|  |  |  | P for trend^c^<.0001 |
| Vitamin B-6 | Q1 | 48/44 | 1 (ref) |
|  | Q2 | 26/66 | 0.32 (0.17-0.61) |
|  | Q3 | 16/77 | 0.19 (0.09-0.38) |
|  | Q4 | 7/85 | 0.06 (0.02-0.17) |
|  |  |  | P for trend^c^<.0001 |
| Vitamin C | Q1 | 46/46 | 1 (ref) |
|  | Q2 | 29/63 | 0.43 (0.24-0.79) |
|  | Q3 | 25/68 | 0.36 (0.20-0.67) |
|  | Q4 | 9/83 | 0.10 (0.05-0.23) |
|  |  |  | P for trend^c^ <.0001 |
| Calcium | Q1 | 69/23 | 1 (ref) |
|  | Q2 | 65/27 | 0.87 (0.45-1.66) |
|  | Q3 | 64/29 | 0.81 (0.42-1.54) |
|  | Q4 | 53/39 | 0.49 (0.26-0.92) |
|  |  |  | P for trend^c^=0.03 |
| Magnesium | Q1 | 54/38 | 1 (ref) |
|  | Q2 | 33/59 | 0.44 (0.24-0.79) |
|  | Q3 | 32/61 | 0.42 (0.23-0.76) |
|  | Q4 | 20/72 | 0.19 (0.10-0.37) |
|  |  |  | P for trend^c^<.0001 |
| Iron | Q1 | 42/50 | 1 (ref) |
|  | Q2 | 30/62 | 0.48 (0.26-0.87) |
|  | Q3 | 29/64 | 0.46 (0.25-0.84) |
|  | Q4 | 18/74 | 0.25 (0.13-0.47) |
|  |  |  | P for trend^c^<.0001 |
| Zinc | Q1 | 26/66 | 1 (ref) |
|  | Q2 | 13/79 | 0.63 (0.35-1.14) |
|  | Q3 | 11/82 | 0.56 (0.31-1.01) |
|  | Q4 | 4/88 | 0.32 (0.17-0.61) |
|  |  |  | P for trend^c^=0.0006 |

BMR, basal metabolic rate; EI, energy intake; GHGE, greenhouse gas emission; CO_2_-eq, carbon dioxide equivalents; DG, Tentative Dietary Goal for Preventing Lifestyle-related Diseases; DRIs, Diet Reference Intakes; EAR, Estimated Average Requirement; RAE, retinol activity equivalent.

^a^ Participants with plausible energy intake was defined as participants with an EI:BMR 1.02-2.35 for men and 1.03-2.36 for women. Participants (184 men and 185 women) were divided into quartiles by usual diet-related GHGE separately by sex, and then combined for analysis. Usual diet-related GHGE was calculated using the Multiple Source Method [43,44] and then adjusted for energy intake by residual method.

^b^ Inadequate intake was defined by comparing usual intake with reference values derived from Dietary Reference Intakes for Japanese 2020 except for iron intake for women under 50 years old and free sugar. For iron intake among women under 50 years old, less than 9.3 mg/d [51] was considered using a probability method. For free sugar, the World Health Organization’s conditional recommendation (<5% energy) was used.

^c^ Logistic regression models were used with the median value in each quartile category of diet-related GHGE as a continuous variable.

# S10 Table**.** Usual food intake (g/d) according to quartile (Q) of usual diet-related GHGE (g CO_2_-eq/day) among 369 Japanese adults (aged 20-69 y) with plausible energy reporting^a^

|  | All (n=369) | Q1 (n=92) | Q2 (n=92) | Q3 (n=93) | Q4 (n=92) | P for trend^c^ |
| --- | --- | --- | --- | --- | --- | --- |
|  |  | 3242 (3000, 3539)^b^ | 3786 (3469, 4077)^b^ | 4074 (3843, 4524)^b^ | 4822 (4398, 5198)^b^ |  |
| Cereals | 450 ± 131 | 480 ± 151 | 462 ± 140 | 429 ± 118 | 430 ± 107 | 0.019 |
| Potatoes | 45 ± 17 | 42 ± 15* | 44 ± 15* | 43 ± 15* | 52 ± 19† | <.0001 |
| Sugar | 15 ± 9 | 14 ± 10 | 14 ± 8 | 17 ± 10 | 15 ± 9 | 0.26 |
| Pulses | 56 ± 33 | 52 ± 37 | 54 ± 27 | 58 ± 34 | 61 ± 35 | 0.25 |
| Nuts | 3 ± 5 | 3 ± 6 | 3 ± 4 | 4 ± 5 | 3 ± 3 | 0.81 |
| Vegetables | 248 ± 91 | 195 ± 64* | 242 ± 75† | 260 ± 82† | 296 ± 108‡ | <.0001 |
| Fruits | 86 ± 77 | 77 ± 78 | 84 ± 73 | 84 ± 67 | 99 ± 87 | 0.26 |
| Mushroom | 15 ± 10 | 12 ± 8* | 13 ± 8*† | 17 ± 11†‡ | 18 ± 12‡ | <.0001 |
| Seaweeds | 5 ± 4 | 4 ± 3* | 5 ± 4*† | 6 ± 4† | 6 ± 4*† | 0.01 |
| Fish and seafood | 41 ± 21 | 32 ± 15* | 41 ± 20† | 43 ± 22† | 48 ± 22† | <.0001 |
| Meat | 95 ± 39 | 86 ± 32* | 91 ± 39*† | 98 ± 41*† | 103 ± 42† | 0.02 |
| Beef | 16 ± 11 | 11 ± 7* | 13 ± 10*† | 17 ± 10† | 23 ± 14‡ | <.0001 |
| Pork | 35 ± 17 | 34 ± 16 | 34 ± 17 | 34 ± 17 | 36 ± 18 | 0.78 |
| Chicken | 31 ± 15 | 31 ± 14 | 29 ± 15 | 33 ± 17 | 30 ± 15 | 0.51 |
| Processed meat products | 13 ± 9 | 12 ± 7 | 14 ± 9 | 13 ± 10 | 11 ± 7 | 0.14 |
| Egg | 40 ± 14 | 40 ± 14 | 39 ± 17 | 39 ± 14 | 41 ± 12 | 0.74 |
| Milk and dairy food products | 100 ± 83 | 94 ± 86 | 120 ± 93 | 89 ± 64 | 97 ± 83 | 0.05 |
| Fat and oils | 21 ± 7 | 22 ± 6* | 23 ± 7* | 20 ± 7*† | 19 ± 5† | 0.0004 |
| Confectioneries | 42 ± 27 | 45 ± 31 | 41 ± 23 | 43 ± 29 | 40 ± 23 | 0.61 |
| Alcohol beverages | 131 ± 222 | 120 ± 218 | 98 ± 154 | 163 ± 232 | 144 ± 266 | 0.21 |
| Tea and coffee | 596 ± 349 | 481 ± 270* | 585 ± 342*† | 621 ± 376† | 698 ± 369† | 0.0003 |
| Sweetened beverages | 38 ± 70 | 53 ± 88* | 35 ± 60*† | 41 ± 80*† | 22 ± 40† | 0.02 |
| Seasonings | 120 ± 66 | 85 ± 33* | 106 ± 47*† | 121 ± 55† | 168 ± 85‡ | <.0001 |
| Water | 506 ± 306 | 438 ± 239* | 506 ± 271*† | 573 ± 373† | 507 ± 314*† | 0.03 |

BMR, basal metabolic rate; EI, energy intake; GHGE, greenhouse gas emission; CO_2_-eq, carbon dioxide equivalents; DG, Tentative Dietary Goal for Preventing Lifestyle-related Diseases; EAR, Estimated Average Requirement; RAE, retinol activity equivalent.

*†‡Maen values within a row with different symbols were significantly different between the quartile group by post hoc Bonferroni’s test (P<0.05).

^a^ Participants with plausible energy intake was defined as participants with an EI:BMR 1.02-2.35 for men and 1.03-2.36 for women. Participants (184 men and 185 women) were divided into quartiles by usual diet-related GHGE separately by sex, and then combined for analysis. Usual diet-related GHGE was calculated using the Multiple Source Method [43,44] and then adjusted for energy intake by residual method. Values are means ± SDs.

^b^ Usual diet-related GHGE (g CO_2_-eq/d): median (25th-75th percentiles).

^c^ Trend of association was examined using a linear regression model with the median value in each quartile as a continuous variable.
