# Supplementary material for: Association between diet-related greenhouse gas emissions and nutrient intake adequacy among Japanese adults
Source: PLoS One. 2020 Oct 23;15(10):e0240803. doi: 10.1371/journal.pone.0240803 (PMC7584234; doi:10.1371/journal.pone.0240803)
Supplement: Attachment — Submitted filename: Response_to_Reviewers_PONE-D-20-15820R1.docx [file pone.0240803.s003.docx]

**Reply to the Editors’ and Reviewers’ comments for the manuscript: PONE-D-20-15820R1**

(Title: Association between diet-related greenhouse gas emissions and nutrient intake adequacy among Japanese adults)

We thank the editor and the reviewers for their very helpful comments on our paper. We have revised the manuscript by addressing each comment point-by-point as described below. All amendments and changes are highlighted in the manuscript using a red font. We trust these changes will resolve any confusion and remedy the shortcomings of the paper.

**Reviewer #1:**

I was a reviewer also of the first version of the manuscript. The authors significantly improved the quality of the manuscript and addressed the previous comments. Only minor revisions remain to be considered before publication.

Authors’ response:

Thank you for your comments.

Introduction

Page 3, line 65-66: The sentence “The dietary aspects have not been included in this statement nor mentioned in the dietary guidelines” is understandable, however it could be slightly modified to increase the readability and clarity by substituting “The dietary aspects” with, for example, “the dietary environmental dimension”.

Authors’ response:

Based on your comments, we have revised the text as follows.

“The environmental dimension of diet has not been included in this statement nor mentioned in the dietary guidelines.” (page 3, line 65-66)

Page 3, line 70-73: the authors should consider to change a bit the sentence to improve the form by substituting “while” (line 71) with “and” or deciding to divide this long sentence in two parts.

Authors’ response:

In accordance with the comment, we have decided to divide this sentence into two parts; one for food consumption and another for nutrient intakes.

“The contemporary Japanese diet is typically high in refined grains, seaweeds, vegetables, fish, legumes and low in whole grains, nuts and seeds, dairy products, sugar-sweetened beverage, and processed and unprocessed red meats [27–29]. At the nutrient level, it is characterized by a high intake of sodium and a low intake of dietary fiber, calcium, and saturated fat [29,30].” (page 3, line 70-73)

Material and methods

Thank you for adding information about the criteria used to determine the sample size and for providing details on the larger study. Thank you also for providing information about data collection and data management. This information is needed for data replicability and study clarity. Furthermore, thank you for adding the post hoc analysis to compare the quartile groups.

Authors’ response:

Thank you for your comments.

Page 7, line 192 and page 8, line 200: Should “quantile” be “quartile”?

Authors’ response:

Thank you for finding our careless typographical errors, which have been corrected as shown below.

“using the median value for each quartile” (page 7, line 195)

“using the median value for each quartile” (page 8, line 200)

“inadequacy were calculated for each quartile of diet-related GHGE” (page 8, line 203)

“^c^ Trend of association was examined using a linear regression model with the median value in each quartile as a continuous variable” (page 16, line 297-298)

Discussion

Page 16, line 316-319: please check the accuracy of the sentence. Meat contribution to diet-related to GHGE has been indicated as 19.6%. This percentage is the highest compared to those referred to the other food groups. As a consequence, meat contribution should not be mentioned together with dairy products (4.6%), but together with cereals (13.1%), vegetables/fruits (7.6%), and fish/seafood (13.8%). Once rectified, the sentence will be compliant with what properly mentioned at page 17, line 334 and 335.

Authors’ response:

In accordance with the comment, we have made the following revision. In line with the revision of the sentences that you pointed out, we have revised some sentences in the same paragraph.

“In Japan, meat was also the top contributor to diet-related GHGE (19.6%), followed by fish/seafood (13.8%) and cereals (13.1%)[11]. Nevertheless, it should be noted that the percentage contribution of meat was lower than that in the Western countries. In addition, the contribution of dairy products (4.6%) was low [11]. In relation to diet-related GHGE, intakes of vegetables, fish/seafood, and meat showed positive associations, while those of cereals and fat and oils showed inverse associations.” (page 17, line 319-323)

Page 18, line 368-370: The authors should mention the lack of objective information on physical activity as a limitation of the study in the discussion section. Indeed, bias can be present in the identification of under- and over- reporters, even though potential effect of energy-misreporting would be small. Another limitation is the relatively limited sample size that should be highlighted.

Authors’ response:

In accordance with the comment, we have added the limitation for sample size and a lack of observational measure for physical activity level.

“Second, our relatively small sample size (n = 396) would limit the power to detect moderate associations with statistical significance. In addition, the estimated usual intake distributions of nutrients and foods would be uncertain due to the small sample size [54]. Nevertheless, significant associations were generally observed between diet-related GHGE and intakes of nutrient and food. Although our sample size might be sufficient to detect the difference between quartiles of diet-related GHGE, further studies with larger sample sizes would be needed. Third,…” (page 17, line 360- page 18, 366)

“Fourth, both nutritional adequacy and diet-related…” (page 18, 368)

“Fifth, due to a lack of objective information on physical activity, bias can be present in the identification of under- and over-reporters. Nevertheless, the potential effect of energy-misreporting in this study would be small.” (page 18, line 378-380)
